# The variations of osseous structure of the ınternal acoustic canal: an anatomical study

**DOI:** 10.1016/j.bjorl.2024.101414

**Published:** 2024-03-05

**Authors:** Elif Sarı, Gkionoul Nteli Chatzioglou, Dastan Temirbekov, Aynur Aliyeva, Adnan Öztürk, Ilke Ali Gürses

**Affiliations:** a Istanbul Aydin University, Faculty of Medicine, Department of Otorhinolaryngology, Istanbul, Turkey; b Istanbul Health and Technology University, Faculty of Medicine, Department of Anatomy, Istanbul, Turkey; c Istanbul University, Faculty of Medicine, Department of Anatomy, Istanbul, Turkey; d The Cincinnati Children's Hospital Medical Center, The Division of the Otolaryngology, Ohio, USA; e Koc University, Faculty of Medicine, Department of Anatomy, Istanbul, Turkey

**Keywords:** Fundus of internal acoustic canal, Internal acoustic meatus, Petrous part, Temporal bone anatomy, Variations of IAC

## Abstract

**Objectives:**

The internal acoustic meatus is an osseous canal that connects the inner ear to the posterior cranial fossa. It is located in the petrous portion of the temporal bone. A thin cribriform osseous plate known as the fundus is situated at the lateral end of the canal. This study assesses the structural and numerical variations of the fundus formations.

**Methods:**

Fifty-four temporal bones of unknown gender and age were examined with the surgical microscope.

**Results:**

The temporal bones analyzed were 46.2% right-sided and 53.7% left-sided. Only one temporal bone had two parallel transverse crests, while three had a single anterior crest that split into two branches posteriorly. The number of foramina at the transverse crest varied, with 29.6% having none, 48.1% having a single foramen, and 22.2% having several foramina. An anterior crest structure was seen in 53.7% of the temporal bones, with 5% having a slightly constricted entry to the facial canal. In cases with a single nerve foramen, 48.1% had one, while 51.8% had more than one, including examples with three or four foramina. A crest was found between the foramina of the single nerve in 7% of patients. Furthermore, a crest between the saccular nerve foramen and the high fiber foramina was seen in 25.9% of cases, and 5% had two saccular nerve foramina.

**Conclusion:**

We think that revealing the anatomical, structural and numerical variations in the fundus will be useful in explaining the disease-symptom relationship.

**Level of evidence:**

Level 4.

## Highlights

• Facial canal crest and double parallel transverse crests are unprecedented findings.
• IAM anatomy variations may explain disease course differences.
• Awareness of anatomical variations in the IAM may help improve surgical outcomes.

## Introduction

The internal acoustic meatus (IAM) is an osseous canal located within the petrous part of the temporal bone, connecting the inner ear to the posterior cranial fossa. It runs laterally within the petrous part and it is approximately 1 cm long. The medial end of the canal is called porus acousticus and enters to the posterior cranial fossa [1]. The lateral end is called fundus which consistst of vertical cribriform osseous plate, where the inner ear and IAM are in contact. The fundus measures an average of 2.5–4.0 mm in height and 2.0–3.0 mm in width. Additionally, the fundus is divided into upper and lower divisions by a transverse crest. The upper half is further divided into anterior and posterior sections by a vertical crest, also called as Bill’s bar. The area of the facial nerve is located in the anterosuperior part of the IAM, while the superior vestibular area is located in the posterosuperior part of the canal [1]. The vestibular nerve (VN) consists of superior and inferior parts. The utriculoampullary nerve, which arises from the intersection of the utricular nerve and the anterior and lateral ampullary nerves, comprises the superior VN and passes through the superior vestibular region. The inferior vestibular area is the transition point of the saccular nerve or ınferior VN [2]. The inferior VN innervates the remaining vestibular structures, the posterior semicircular canal, and the saccule, whereas the superior VN innervates the superior and lateral semicircular canals, as well as the utricle [3]. The cochlear area, located in the lower and anterior part of the fundus of the IAM, is the passageway of the cochlear nerve fibers passing through the modiolus of the cochlea [2]. The singular canal is situated at the level of the fundus, between the ampulla of the posterior semicircular canal and the inferior part of the IAM.The posterior ampullary nerve passes via this canal.

The fundus of the IAM shows wide anatomical variations [2], [4], [5]. In this study, we aimed to evaluate the anatomical structures of the bony fundus, as well as to outline structural and numerical differences of the formations in the fundus.

## Methods

The study was conducted following the approval of the Clinical Research Ethics Committee of Medicine Faculty (Date: 07.05.2019; Number: 627). Fifty four temporal bones of unknown gender and age, used for educational purposes in the bone collection of Istanbul University, Faculty of Medicine, Department of Anatomy were examined. Fundus of all temporal bones was examined with the ×40 magnification of the surgical microscope (DVF D.F. Vasconcellos S.A. São Paulo, Brasil). The crests separating the nerves and the foramina belonging to the nerves were evaluated structurally and numerically.

## Results

**Fig. 1:**
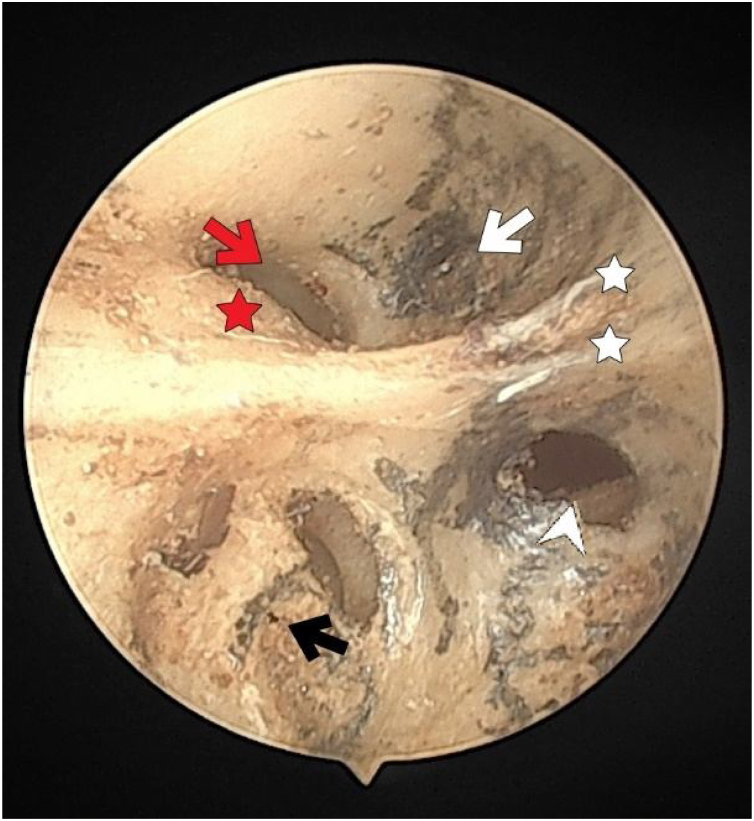
The fundus of internal acoustic meatus (view from medial side of the right temporal bone). Cochlear nerve foramen (black-arrow); the buldge of facial canal (red-star); canals for superior vestibular nerve (white-arrow); transverse crest (white-star); facial canal (red-arrow); inferior vestibular nerve foramen (white-arrowhead).

The structural and numerical characteristics of foramina of the IAM are summarized in Table 1. Of the 54 temporal bones used in the study, 25 (46.2%) were right and 29 (53.7%) were left temporal bones. The fundus was divided into quadrants of different sizes by numerous vertical and transverse crests. The facial nerve foramen, superior VN foramen, cochlear nerve foramen, and inferior VN foramen were all situated in the anterosuperior, posterosuperior, anteroinferior and posteroinferior quadrants, respectively (Fig. 1).

**Table 1:** The structural features of crests and foramen/foramina numbers in the fundus of internal acoustic meatus.

The fundus of the internal acoustic meatus		n
Anterior crest		29
Crest at facial nerve entry		3
Bump at the facial nerve entry		2
Crest in the foramen of the superior vestibular nerve		30
Number of transverse crest foramen	0	16
	1	26
	>1	12
Number of singular nerve foramen	1	26
	2	24
	3	2
	4	2
Crest between saccular nerve foramen and high fiber nerve foramina		14

**Fig. 2:**
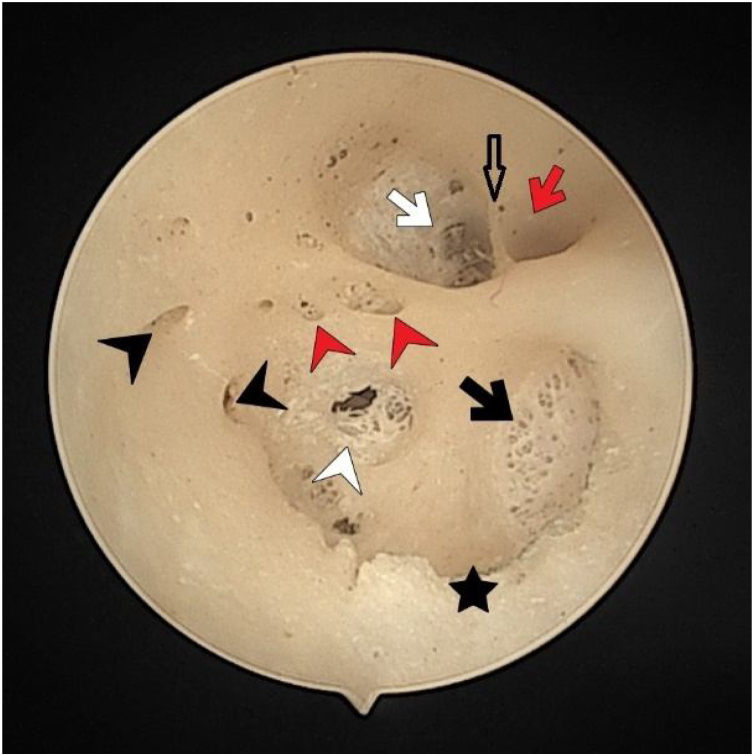
The fundus of internal acoustic meatus (left temporal bone). Facial canal (red-arrow); canals for singular nerve (black-arrowhead); saccular nerve foramina (white-arrowhead); canals for superior vestibular nerve (white-arrow); anterior crest (black-star); foramen of the transverse crest (red-arrowhead).

The vertical crest (Bill’s bar) was located more medially with no detected structural differences (Fig. 2). On the other hand, it was observed that the transverse crests showed more parabolic arrangement rather than a straight extension. There were 2 transverse crests parallel to each other in one of the temporal bones (0.1%). In three cases (0.05%), it started as a single crest anteriorly and ended by dividing into 2 different branches posteriorly (Fig. 1). These cases had a transverse crest foramina between the arms of the transverse crest (Fig. 1). In all cases, the transverse crest foramina were located laterally and their number varied.

**Fig. 3:**
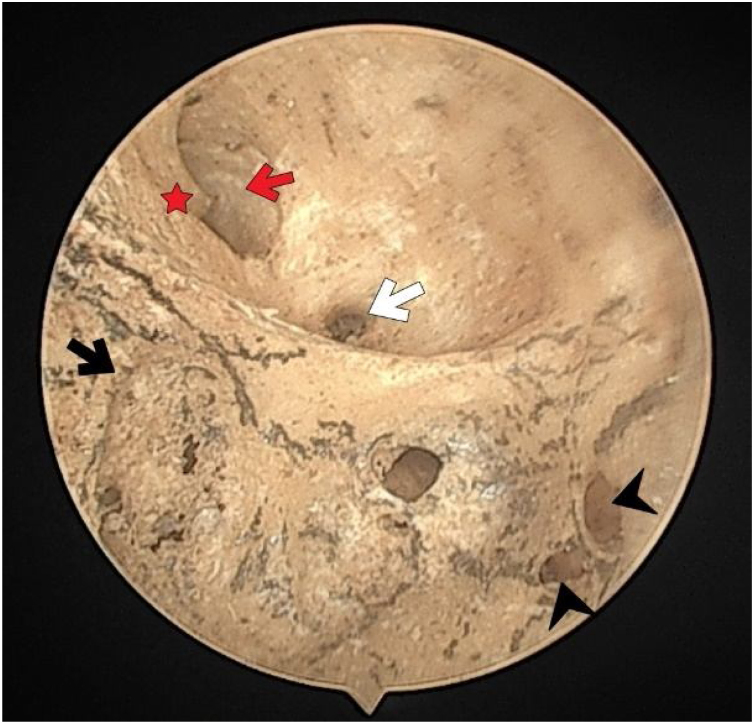
The fundus of internal acoustic meatus (right temporal bone). Crest of Facial canal (red-star); facial canal (red-arrow); Foramina for cochlear nerve (black-arrow); canals for singular nerve (black-arrowhead); canals for superior vestibular nerve (white-arrow).

**Fig. 4:**
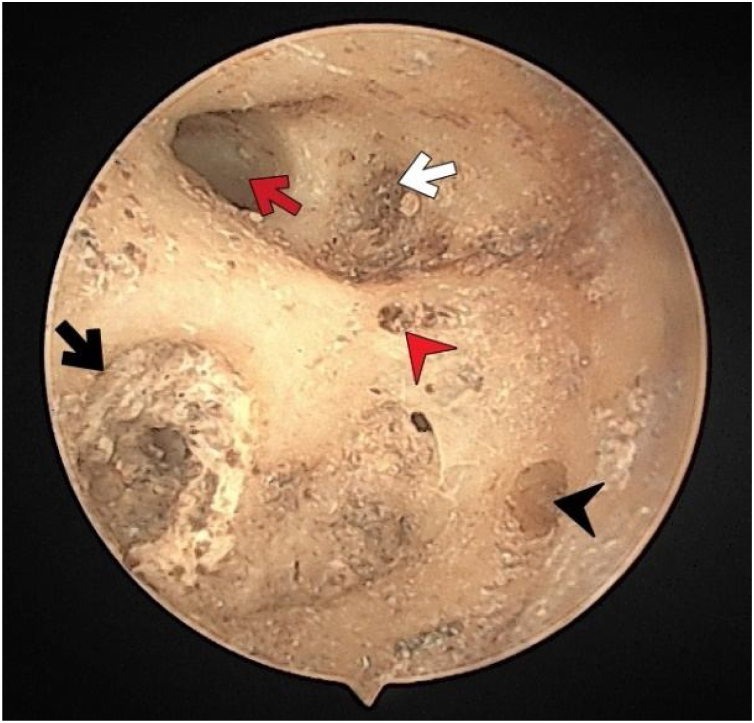
The fundus of internal acoustic meatus (right temporal bone). Facial canal (red-arrow); foramen of the transverse crest (red-arrowhead); canals for superior vestibular nerve (white-arrow); foramina for cochlear nerve (black-arrow); canals for singular nerve (black-arrowhead).

There were no foramen at the transverse crest in 16 (29.6%) specimens (Fig. 3), a single foramen was present in 26 (48.1%) (Fig. 4), and more than 1 foramina in 12 (22.2%) bones (Fig. 2). In specimens with multiple foramina, 1 foramen was more prominent than the others. In samples without a transverse crest foramen, a foramen was usually seen above the crest, under the superior VN foramen, or below the crest, and above the saccular nerve foramina.

More than half of the temporal bones (29 of the 54) (53.7%) were found to have an anterior crest structure (Fig. 2). When present, it was located on the anteroinferior wall at the entrance of the IAM and partially narrowed the view of the cochlear foramen when looking from the porus.

The facial nerve foramen was located at the anterosuperior quadrant. In three cases (5%), there was a crest anterior to the facial canal that partially narrowed its entrance (Fig. 3). In two cases (3%), a bony hump was present, instead of a crest, at the entrance of the foramen (Fig. 1).

The cochlear nerve canal was in the form of a cribriform plate at the anteroinferior quadrant, close to the cochlea. Since this cribriform plate has a very thin bone structure, it was observed as a single wide opening in some bones due to crush.

The superior VN foramen was located at the posterosuperior quadrant. A horizontally extending crest was detected in 30 (55.5%) of the cases. One of them had 2 horizontally extending crests.

The singular nerve foramen was observed in the posterior wall of the IAM. It was located a little far from the fundus. While 26 (48.1%) cases had 1 foramen (Fig. 4), 28 (51.8%) had more than one foramen (Fig. 2, Fig. 3). Of these, it was observed that there were 3 foramina in 2 cases and 4 in 2 cases. One of the foramina had a larger diameter than the other foramina. There was a crest between the foramen of the singular nerve in 4 cases (7%).

The saccular nerve foramen was located superior to the high fiber nerve foramina and occurred as a cribriform plate. In some specimens the cribriform plate was deformed due to its fragile structure and was observed as a single wide opening. A crest was observed between the saccular nerve foramen and high fiber foramina in 14 (25.9%) cases. Three specimens (5%) had 2 saccular nerve foramina.

## Discussion

When evaluating inner ear pathologies, knowing the anatomy of the IAM is of great importance both in the evaluation and management of the diseases of this region and in the planning of surgery for this region. Good knowledge of anatomy allows a better interpretation of imaging techniques and reveals the disease-symptom relationship while minimizing iatrogenic damage during surgery. The first study on the fundus of the IAM was done by Fatterpaker et al. They evaluated the topography and morphology of the channels in the fundus with CT scanning and reported the results of their measurements [6]. In our study, the examination of the fundus with the aid of a microscope allowed a more detailed evaluation of the structural differences of the fundus and the visualization of the smaller foramina. The IAM fundus has wide anatomical variations [5].

As described by Kozerska and Skrzat, the transverse crest showed a parabolic extension contrary to the straight line that appears in most of the schematic drawings [2]. In one of the cases, the transverse crest extended as 2 parallel crests. In all 3 cases, the transverse crest started alone in the anterior and ended in 2 separate branches posteriorly. No publications describing these structures were found in the literature. Some studies have shown that foramina may exist in the transverse crest and these foramina terminate in different regions according to the location of the transverse crest [5]. It has been demonstrated that foramina located at or above the upper side of the crest end in the utriclar macula and those that located under of the crest end in the saccule [5]. In the study carried out by Kozerska and Skrzat the transverse crest foramen was identified in only infant skulls. They hypothesized that it leads to the vestibule [2]. The anterior vestibular artery, or a branch of it, which is the first branch of the labyrinthine artery, was considered to be present in the canal, which Mei et al. described as the transverse crest foramen in all of their samples [7]. In our study, only adult temporal bones were evaluated and 86.3% of them were found to have at least 1 foramen. In bones with more than one foramen, one foramen was observed to be larger than the others. It has been suggested that these foramina contain otolith organs, blood vessels, or nerve branches that supplied the vestibular wall or superior vestibular canal [2], [5]. We also thought that these foramen transmit blood vessels and nerve branches. We speculated that there may be more than one foramen in bones with more arterial vessel supply. In conditions like Meniere's disease, age-related hearing loss (presbyacusis), abrupt hearing loss, sudden hearing loss due to noise exposure, and tinnitus, a reduction in cochlear microcirculation is acknowledged as an etiological component [8], [9], [10], [11], [12], [13], [14], [15], [16], [17], [18], [19]. We hypothesized that patients who have more than 1 transverse crest foramen could have less risk of diseases that may occur due to insufficient cochlear microcirculation. In the bones that lack a transverse crest foramen often there is foramen at the superior VN canal's base or above the saccular nerve foramina [5]. These foramina were seen in some of the bones without transverse crest foramen in our study too.

Of the 54 cases, 2 had a hump and 3 had a crest at the anterosuperior aspect of the IAM near to fundus that narrows the entrance of the facial nerve foramen. No previous study has reported such a structure in the literature. Accordingly there is no data about if these crests and/or humps have any clinical significance. We believe that the clinical and prognostic effects of these crests and humps in inflammatory, ischemic and neoplastic pathologies of the IAM need to be investigated.

The superior VN canal is the passageway for the utricular nerve, the lateral and superior ampullary nerve, and both. The utricular nerve usually runs inferiorly, sometimes separated by a prominent crest [5]. In our study, there was a crest that we think separates the utricular nerve from the lateral and superior ampullary nerves in 55.5% of the specimens. In one of the temporal bones, the superior VN canal was divided into 3 sections with 2 horizontal crests.

Some surgical treatments, like transmeatal transcochlear cochleovestibular neurectomy and retrosigmoid acoustic neuroma surgery, utilize the solitary nerve foramen as a marker [20], [21]. It is located on the posterior wall of the IAM. In a study conducted by Schart-Morén et al., it was reported that 2 of 324 samples (0.6%) had two ducts of similar size that merged before reaching the ampulla [5]. In our study, we observed that 51.8% had more than one channel. Since it was observed with a microscopic view, it was not possible to distinguish whether the canals were a blind canal or not, whether they merged during their course and where they terminated. As in the transverse crest foramen, one canal had a larger diameter than the others in bones with more than one canal. We thought that the posterior ampullary nerve passed through the large-diameter foramen, while the other small foramina either ended as a blind canal or were followed by blood vessels. The defect in the inner ear microcirculation affects both the auditory and vestibular organs. We hypothesized that inner ear pathologies would be less common since arterial microcirculation would be better in cases with blood vessels passing through the foramen.

The saccular nerve had a cribriform plate located below the transverst crest foramen, superior to the high fiber nerve foramina. In some bones, this cribriform structure was observed as a single opening because of deformation. As in previous studies, we observed that there is a crest between the saccular nerve foramen and the high frequency nerve foramen [5]. This crest structure was present in 14 of 54 cases. It has been reported in the literature that, although rare, there may be two saccular nerve canals [5]. In our study, it was observed that only 3 of 54 cases had 2 different nerve canals.

The cochlear nerve canal was in the form of a cribriform plate close to the cochlea. Since this cribriform plate has a very thin bone structure, it was observed as a single, wide opening in some bones. The anterior wall of the IAM was angled anteriorly to partially cover the cochlear canal when viewed from the opening. This structure, called the anterior crest, was detected in more than half of the our samples. No numerical data were found in the literature regarding the presence of anterior crest.

## Conclusion

We think that revealing the anatomical, structural and numerical variations in the fundus will be useful in understanding why the course of the diseases varies among individuals and explaining the pathophysiological variations and disease–symptom relationship. In addition, knowing the anatomical variations will increase the surgical success in order to minimize iatrogenic damage during the surgery of the region.

## Author contributions

All authors contributed to the creation and revision of the manuscript. All authors read and approved the final manuscript.

## Funding

Authors have not have got any funding related to the manuscript.

## Ethics approval and consent to participate

Ethics approval was obtained from the Clinical Research Ethics Committee of Istanbul University, Faculty of Medicine (Date: 07.05.2019; Number: 627).

## Conflicts of interest

The authors declare no conflicts of interest.
